# Long-term metabolic consequences of *in-utero* gestational diabetes exposure and maternal treatment interventions: a systematic review and meta-analysis

**DOI:** 10.3389/fendo.2026.1904624

**Published:** 2026-09-16

**Authors:** Yufeng Wu, Ke Shen, Huihui Guo, Hongjing Wang

**Affiliations:** 1 Department of Gynecology and Obstetrics, West China Second University Hospital, Sichuan University, Chengdu, Sichuan, China; 2 Key Laboratory of Birth Defects and Related Diseases of Women and Children (Sichuan University), Ministry of Education, Chengdu, Sichuan, China

**Keywords:** dyslipidemia, GDM, gestational diabetes mellitus, glycaemic control, HbA1c, HOMA-IR, insulin resistance, *in-utero* exposure

## Abstract

**Background:**

Gestational diabetes mellitus (GDM) affects 14–17% of pregnancies globally, imposing long-term public health burdens beyond pregnancy. Maternal hyperglycaemia increases offspring risk of metabolic disorders through epigenetic, hormonal and developmental programming. Growing evidence connects prenatal GDM exposure to childhood and adult metabolic diseases, yet the consistency and magnitude of these associations across different metabolic conditions remains unclear.

**Methods:**

We systematically searched PubMed, Cochrane CENTRAL and Embase. Eligible studies included observational cohorts exploring associations between *in-utero* GDM exposure and offspring metabolic outcomes, and RCTs assessing maternal antenatal interventions. Bias risk was evaluated with Cochrane RoB 2 (for RCTs) and the Newcastle-Ottawa Scale (NOS) (for observational studies). Separate outcome-based pooled analyses were conducted, given the distinct clinical questions addressed by the two study designs. All meta-analyses used the random-effects inverse-variance model. Heterogeneity was assessed via I² and Cochran’s Q statistic, and Egger’s linear regression test was used to detect publication bias.

**Results:**

Sixteen studies (n=9 RCTs, n=7 observational cohorts) involving diverse populations across 11 countries were included. In observational cohorts, GDM-exposed offspring showed higher BMI (MD = 1.42 kg/m2, 95% CI: 0.94-1.89) and greater estimates for overweight/obesity in adulthood (OR = 2.09, 95% CI: 1.25–3.50, I² = 80.7%). The analysis of fasting glucose results displayed high variability (I² = 80.7%) yet produced no meaningful combined impact (MD = -0.016 mmol/L, 95% CI: -1.52 to 1.49). In RCTs, active antenatal GDM management was associated with a non-significant trend towards lower HOMA-IR (MD = -0.30, 95% CI: -0.61 to 0.01; I² = 68.2%) and HbA1c (MD = -0.41%, 95% CI: -0.85 to 0.03; I² = 99.1%) relative to usual care. There were no statistically significant combined effects found for 2-hour OGTT glucose levels total cholesterol levels triglyceride levels or HDL-C levels. Egger’s test revealed no significant publication bias for either glucose (t = 0.82, p = 0.43) or lipid outcomes (t = -0.08, p = 0.94).

**Conclusion:**

*In-utero* GDM exposure confers metabolic risks from childhood to adulthood; lifelong persistence requires verification via extended longitudinal research GDM interventions including diet, exercise and pharmacotherapy may lower offspring risk, although heterogeneity necessitates cautious interpretation.

## 1 Introduction

Gestational diabetes mellitus (GDM) exists as a medical condition which causes glucose intolerance to develop in women who become pregnant. The current worldwide prevalence of GDM amongst pregnant women reaches 14 to 17% because people now face more obesity problems and they exercise less and women have children at later ages according to the International Diabetes Federation (1). The medical community considers GDM to be a temporary condition of pregnancy which ends after a woman gives birth but current research shows that GDM creates metabolic effects that last until death and directly affect the health of the child.

The developmental origins of health and disease (DOHaD) theory argues that negative fetal development environments induce physiological changes which lead to chronic non-communicable diseases that appear during adult life (2, 3). Maternal hyperglycaemia during GDM exposure was associated with developing foetuses to experience excessive glucose levels which result in fetal hyperinsulinaemia and pancreatic beta-cell development changes and fat tissue production development and permanent alterations to metabolic gene regulatory networks (4). The researchers believe that these intrauterine disturbances create a condition which develops into obesity and insulin resistance and cardiometabolic problems that persist from childhood through adulthood without any impact from postnatal lifestyle choices.

GDM exposure during pregnancy increases the likelihood of offspring developing obesity, glucose tolerance, type 2 diabetes mellitus (T2DM), hypertension, and dyslipidaemia (5, 6). The EPOCH study established that GDM pregnancy offspring developed obesity and metabolic fasting glucose disorders at higher rates than children born to mothers with normal blood sugar levels who belonged to the same Pima Indian community (7). The ALSPAC birth cohort study reported that GDM offspring in a European population showed higher rates of childhood obesity together with increased risk markers for cardiometabolic disease. The research indicates that exposure to hyperglycaemia during pregnancy will create permanent beta cell damage and liver fat buildup as well as changes in adipokine production amongst the offspring (8).

Emerging evidence suggests that maternal hyperglycaemia can lead to persistent epigenetic changes, oxidative stress, mitochondrial dysfunction, and chronic low grade inflammation, plus altered placental nutrient transport, that maybe contributes to long term metabolic programming in the offspring. Existing evidence has been significant yet essential research areas have remained unexplored.

The current research fails to produce consistent findings because it uses different methods to diagnose GDM according to IADPSG guidelines, Carpenter-Coustan, WHO (1999 - 2013) standards, and NDDG guidelines whilst assessing outcomes at different times, measuring various metabolic effects, and controlling different levels of confounding variables. The research lacks complete evidence because it has not yet examined all available studies about how GDM treatment methods during pregnancy which include dietary changes and exercise and metformin use and insulin administration have affected metabolic health in offspring. Previous systematic reviews have concentrated on specific results or have failed to include data from newly released randomized studies which evaluated cardiometabolic results in offspring as secondary outcomes.

This systematic review and meta-analysis presents how GDM pregnancies affect metabolic health in their children over their entire lives, and analysed combined effects through its main metabolic domains, which included glycaemia measurements, body fat assessment, and lipid testing, along with blood pressure evaluation. Therefore, the main objective of this systematic review and meta-analysis was to synthesize the evidence about (i) long term metabolic consequences in offspring with *in-utero* exposure to GDM, and (ii) whether maternal treatment of GDM during pregnancy influences offspring outcomes.

## 2 Methods

The systematic review and meta-analysis followed the PRISMA 2020 standards for the identification of literature studies according to these guidelines (9).

### 2.1 Eligibility criteria

The PICOS framework for this systematic review and meta-analysis is presented as follows, where the eligible studies were categorized in two pre-defined evidence:.

Population (P): Offspring (infants, children, adolescents, or adults) of pregnant women with GDM-affected pregnancies, compared to offspring of normoglycaemic pregnancies. Studies included all participants regardless of their gestational age or their ethnicity or their parity status.

Intervention/Exposure (I): *In-utero* exposure to GDM, defined by any validated diagnostic criterion including IADPSG (fasting plasma glucose ≥5.1 mmol/L, 1-hour ≥10.0 mmol/L, or 2-hour ≥8.5 mmol/L on 75g OGTT), Carpenter-Coustan, WHO 1999, or NDDG thresholds.

*Comparator (C):* Offspring of normoglycaemic pregnancies who used matching or non-matching methods. The RCT subanalyses allowed researchers to compare GDM treatment arms which included lifestyle intervention and standard care whilst evaluating metabolic outcomes of their offspring.

Outcomes (O): Primary outcomes: (1) BMI or BMI z-score; (2) overweight/obesity prevalence; (3) fasting plasma glucose; (4) 2-hour OGTT glucose; (5) HbA1c; (6) HOMA-IR or fasting insulin. Secondary outcomes: waist circumference measurements, body fat percentage, systolic blood pressure, and diastolic blood pressure along with total cholesterol measurements, LDL-C, HDL-C, triglyceride, and metabolic syndrome prevalence assessments.

Study Design (S): Observational cohort studies (prospective and retrospective), case-control studies, and randomised controlled trials which all occurred in peer-reviewed English-language journals between January 2000 and June 2026.

Since observational studies suggested the link between *in utero* exposure to GDM and metabolic outcomes in offspring, whilst RCTs focused on maternal interventions during pregnancy, these types of evidence were analysed separately. Quantitative synthesis was basically done by outcome, but the conclusions made a clear distinction between exposure related associations, and effects that came from the interventions. Therefore, this review addresses two related but methodologically distinct questions.

Strand A (Exposure). P: Offspring of GDM-affected pregnancies. E: *In-utero* GDM per any validated diagnostic criterion (IADPSG, Carpenter-Coustan, WHO 1999, NDDG). C: Offspring of normoglycaemic pregnancies. S: Observational cohort or case-control studies only.

Strand B (Treatment). P: Offspring of GDM-affected pregnancies. I: an active antenatal treatment for GDM (dietary/lifestyle, exercise, pharmacological, or early screening/prevention). C: standard care or placebo within the same trial. S: randomized trials only.

Studies were excluded if they: (1) were animal studies, case reports, or narrative reviews; (2) did not report original data; (3) examined only maternal outcomes without offspring data; (4) lacked a comparator group of non-GDM offspring; (5) were conference abstracts without full-text publications; or (6) were published in languages other than English.

### 2.2 Search strategy

A complete electronic search was performed across three databases which included PubMed National Library of Medicine (NLM), Cochrane Central Register of Controlled Trials (CENTRAL), and Embase. The search included all publications that were published between January 2000 and June 2026. The search strategy was defined on purpose to include those studies that comprehend two complementary parts of the research question. The search strategy combined MeSH terms and free-text keywords across three conceptual domains: (1) gestational diabetes exposure, (2) offspring/paediatric population, and (3) metabolic outcomes. The complete search strategy is detailed in **Supplementary Tables 1**, **2**. The final PubMed search resulted in 5726 records which were obtained through the query “Diabetes, Gestational”[Mesh] OR gestational diabetes[tiab] AND offspring/children/adolescent AND metabolic outcomes which filtered for Abstract and Full text and Clinical/Observational Study and English and Humans and 2000–2026. The Cochrane CENTRAL search produced 459 records whilst Embase provided 228 records which resulted in 6413 total records from all databases. The researchers conducted a manual search of reference lists from included studies and relevant review articles to find additional studies that met eligibility criteria. The research did not include any searching of grey literature.

### 2.3 Study selection

All identified records were imported into a reference management system (Endnote 21) which led to the removal of 400 duplicate records that resulted in 6,013 unique records for screening. Two reviewers (SI and co-reviewer) conducted independent title and abstract screenings to determine which materials matched the inclusion/exclusion criteria. During full-text screening, the studies were classified study design and research objective before the data extraction, so that the observational and interventional evidence could be interpreted separately. ANy disagreement amongst the reviewers were resolved either through discussion or third-party adjudication. Lastly, 1,029 initial screened records were evaluated for their eligibility status, and the final inclusion comprised of 16 studies which all qualified for both qualitative synthesis and meta-analysis. The complete study selection procedure appears in the PRISMA 2020 flow diagram (**Figure 1**).

**Figure 1:**
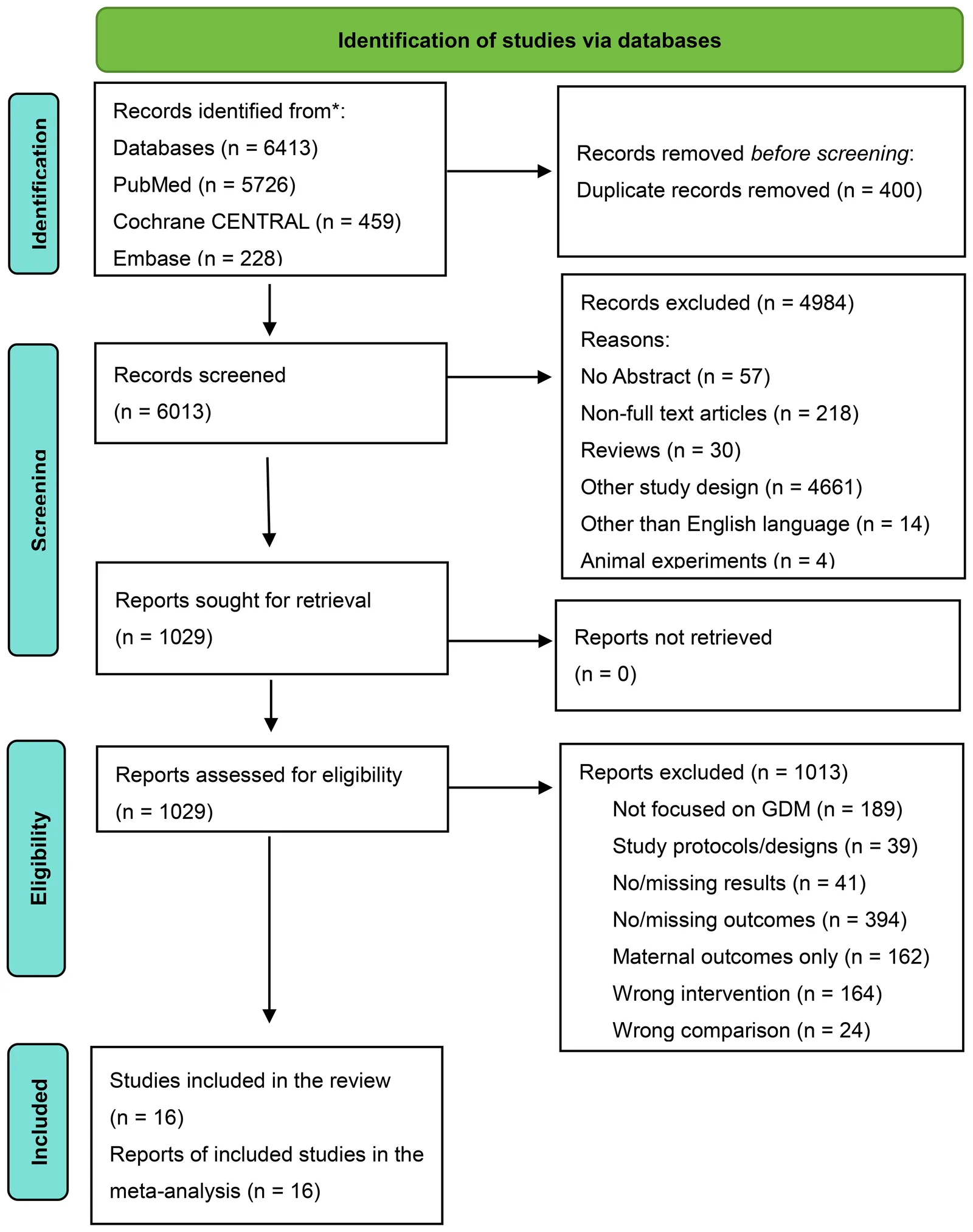
PRISMA flowchart diagram, where ‘n’ is the number of studies.

### 2.4 Data extraction

A standardized data extraction form was created. Two reviewers independently extracted the following data from each included study: (1) bibliographic details (author, year, country, setting, study design, recruitment period, follow-up duration); (2) population characteristics (sample size, offspring age, maternal age, pre-pregnancy BMI, parity, GDM diagnostic criteria, gestational age at diagnosis, treatment received); (3) outcome data with measures of central tendency and dispersion [mean ± SD, or median (IQR)] for each metabolic outcome, alongside effect measures (MD, OR, RR, HR) and 95% confidence intervals (CI); and (4) quality assessment scores. The research team reached out to corresponding authors for missing information. Two reviewers independently collect data into a structured extraction database (Microsoft Excel) which a third reviewer verified.

### 2.5 Quality assessment

The researchers evaluated methodological quality through established assessment tools which fitted each study method they used. The Cochrane Risk of Bias 2 (RoB 2) tool assessed randomised controlled trials through five bias evaluation domains which included randomisation process (D1), deviations from intended interventions (D2), missing outcome data (D3), measurement of the outcome (D4), and selection of the reported result (D5) (10). The assessment of overall risk of bias was conducted through three categories which included Low and Some Concerns and High. The Newcastle-Ottawa Scale (NOS) evaluated observational cohort studies and retrospective studies through its assessment of selection which used four items and comparability which used two items and outcome assessment which used three items to create a maximum score of 9 stars. The NOS classification system assigned low risk to scores between 7 and 9 and moderate risk to scores between 4 and 6 and high risk to scores between 0 and 3.

### 2.6 Statistical analysis

The meta-analyses in R version 4.5.3 was performed through the meta package. This research provided estimates for both a common-effects and random-effects model which used restricted maximum-likelihood (REML) estimation method to calculate between-study variance (tau²) for studies which displayed both clinical and methodological differences. The summary effect measure combined two types of assessments which used mean difference (MD) and 95% confidence intervals for continuous outcomes and odds ratio (OR) with 95% confidence intervals (CI) for binary outcomes. Outcomes that were continuous and contain different units were changed into common units first, before any quantitative synthesis, whenever it felt necessary. Also all reported effect estimates along with their units were double-checked against the original publications to make sure consistent reporting of the estimates. The I² statistic and Cochran’s Q-test were used to measure heterogeneity whilst I² values established three levels of heterogeneity which were interpreted as low (less than 25%) moderate (25 to 75%) and high (above 75%) (11). The Q-profile method was used to calculate 95% CI for tau². Egger’s linear regression test was used to evaluate publication bias for meta-analyses which included at least 10 studies in the combined glucose and lipid pools. The funnel plots were illustrated to show publication bias through visual examination. A pre-defined subgroup analyses was also conducted based on study design (RCT vs. observational), intervention type (dietary/lifestyle, pharmacological, exercise), and offspring age at outcome assessment. A sensitivity analysis was also performed which removed studies displaying high risk of bias. Although pooled analyses were done based on individual metabolic outcomes, the observational and interventional evidence was interpreted individually, because different study designs address different clinical questions. The statistical significance was set at when the two-sided p value reached a threshold of 0.05 or lower.

## 3 Results

### 3.1 Study selection

The database search produced 6413 records which included PubMed (n = 5726), Cochrane CENTRAL (n = 459), and Embase (n = 228). After removing 400 duplicates, 6,013 records were screened at the title/abstract stage. Of these, 4,984 records were excluded: 57 lacked abstracts, 218 were not available as full-text articles, 30 were narrative or systematic reviews, 4,661 used other study designs not meeting inclusion criteria, 14 were non-English, and 4 were animal studies. About 1,029 research studies were assessed for eligibility and excluded 1,013 reports which contained the following reasons for exclusion: not focused on GDM (n = 189), study protocols without results (n = 39), no or missing results (n = 41), absence of eligible metabolic outcomes (n = 394), maternal outcomes only without offspring data (n = 162), wrong intervention or comparator (n = 188). Sixteen studies (12–27) met all eligibility criteria and were included in the final systematic review and meta-analysis.

### 3.2 Characteristics of included studies

Sixteen research studies which were published between 2009 and 2025 that contained 9 RCTs and 7 observational studies which included four prospective population-based and three retrospective cohort studies with audit studies (**Table 1**). The research studies took place in different countries. The studies reported a range of sample sizes within 34 and 5043 participants. The researchers followed participants from infancy until they reached early adulthood, whilst their median follow-up period in observational studies lasted between 7 to 10 years. The studies reported maternal age as the average between 27 and 35 years, whilst pre-pregnancy BMI showed a range from normal weight to obese categories in all studies.

**Table 1:** Characteristics of the included studies.

Study							Population				GDM diagnosis					Quality assessment	
Author/year	Design	Country	Setting	Period/follow-up	Sample size (I/C)	Study category	Age	Maternal age	Parity	Pre-pregnancy BMI	Diagnostic criteria (IADPSG, carpenter-coustan, WHO, NDDG)	Screening criteria (OGTT, GCT)	Gestational age at diagnosis/start of intervention	Fasting glucose	Treatment (insulin, metformin, glyburide, diet/lifestyle)	Risk	Quality
25	Observational cohort	China	Multicentre	July 2017-Dec 2018	3172 women	Exposure	20-40	NR	NR	NR	19% GDM	NR	28–42 weeks gestation	NR	NR	Low RoB	High
20	Randomized, parallel, controlled, double-blind trial	England	Multicentre	Nov 2019-July 2023	428 (214/211)	Treatment (diet/lifestyle/exercise) vs usual care	≥ 18	33.03 (5.04)	35% primiparous	25–30 kg/m2	NR	OGTT	22.85 (6.40)	NR	diet/lifestyle	Low RoB	High
23	RCT	Norway	Multicentre	2007-2009	118	Treatment (diet/lifestyle/exercise) vs usual care	31.2 (3.6)	NR	52% nulliparous	23.1 (3.0)	NR	OGTT	33.7 (2.0)	NR	diet/lifestyle	Some concerns	Moderate
13	Population-based cohort	Qatar	Multicentre	Jan 2017-Dec 2017	1260/3783	Exposure		31.59 (5.87)	NR	33.80 (8.31)	NR	NR	38.12 (2.04) vs 38.34 (2.57)	NR	GDM	Some concerns	Moderate
22	Retrospective review	Canada	Multicentre	Jan-Dec 2007	294 (55/239)	Exposure	29.3 (4.7)	NR	55.1% nulliparous	25.5 (6.2)	NR	NR	NR	6.67 (1.88)	GDM	Some concerns	Moderate
24	Observational prospective follow-up	NICHD	Medical centre	Feb 2012-Sep 2013	981 (56)	Exposure	7 (6-8)	NR	NR	NR	NR	NR	NR	NR	NR	Some concerns	Moderate
19	Pilot or feasibility RCT	USA	Medical centre	April 2010-April 2012	68 (33/35)	Treatment (diet/lifestyle/exercise) vs usual care	18-40	> 29 years: 24.2%	19.4% nulliparous	45.5% overweight	NR	NR	NR	NR	diet/lifestyle	Some concerns	Moderate
17	Observational retrospective cohort	Argentina	Primary care centres and maternity hospitals	2017-2019	1870 recruited	Exposure	30.7 (6.7)	29.3 (5.8)	2.2 (2.0)	NR	NR	NR	NR	NR	NR	Some concerns	Moderate
18	PregMet RCT	Norway	Multicentre	Feb 2005-Jan 2009	257 pregnant women (71/70)	Pharmacological (metformin)	29.5 (3.9)		62% nulliparous	29.0 (6.9)	21% GDM in Mgroup	NR	5–12 weeks gestation	4.6 (0.5)	Metformin	Some concerns	Moderate
26	Lifestyle in Pregnancy (LiP) RCT	Denmark	Hospital	Oct 2007-Oct 2010; 304 followed till delivery	360 obese women (150/154)	Treatment (diet/lifestyle/exercise) vs usual care	29 (27-32)	NR	52.7% primiparous	30–45 kg/m2	NR	NR	28–30 weeks gestation	NR	diet/lifestyle	Some concerns	Moderate
15	Retrospective audit	Australia	Teaching hospital	2004	136 GDM	Exposure	≥ 30	30.7 (5.7)	NR	NR	43 GDM	OGTT	28–37 weeks gestation	NR	NR	Some concerns	Moderate
12	RCT	USA	Prenatal care clinic	April 2018-March 2020	34	Treatment (diet/lifestyle/exercise) vs usual care	27 (5.3)	NR	29 nulliparous	35 (4.2)	56% GDM	GCT	< 20 weeks gestation	NR	diet/lifestyle	Low RoB	High
27	RCT	China	Hospital	Dec 2014-July 2016	300 singleton women (150/150)	Treatment (diet/lifestyle/exercise) vs usual care	32.1 (4.57)	NR	NR	26.78 (2.75) kg/m2	22% GDM in exercise group	OGTT	10 weeks gestation	NR	Exercise	Low RoB	High
21	Secondary analysis of pragmatic RCT	Washington	Healthcare system	May 2015-Oct 2019	326 (89/79)	Prevention/screening	31.3 (3.5)	NR	NR	34.8 (5.8)	73% GDM	NR	NR	NR	Early GDM screening	Low RoB	High
16	Open-label, parallel RCT	Italy	Hospital	July 2013-Oct 2015	80 GDM women (20 per arm)	Pharmacological (inositol)	18–45 years	35.0 (4.4)	20% nulliparous	25% overweight	75 g oral glucose tolerance test (OGTT)	OGTT	24–28 weeks of gestation	88.5 (7.3)	Myo-inositol + D-Chiro-inositol	High RoB	Low
14	Observational cohort	Denmark	Multicentre	2003-2005	1066 offspring of women with GDM	Exposure	21.6 (1.8)	29.5 (5.4)	58% multiparous	40% overweight (>25)	NR	OGTT	273 (247-284)	NR	NR	Some concerns	Moderate

GDM, gestational diabetes mellitus; GCT, glucose challenge test; RCT, randomized controlled trial; RoB, risk of bias; OGTT, oral glucose tolerance test; NR, not reported; USA, United States America.

The studies used different GDM diagnostic criteria because six studies adopted OGTT-based criteria which followed IADPSG, WHO, and Carpenter-Coustan thresholds, whilst three studies implemented oral glucose challenge test (GCT) screening, and seven studies either failed to state their criteria, or they followed their institutional protocols. The RCTs studied various therapeutic interventions through different methods, which included seven trials on dietary and lifestyle changes, one trial on exercise programs, one trial on metformin, one trial on myo-inositol plus D-chiro-inositol supplementation, and one trial on pre-pregnancy weight loss combined with early GDM screening. The observational studies evaluated outcomes from GDM pregnancies by studying their offspring, whilst they chose not to disclose the specific GDM treatment details that occurred during the index pregnancy. Moreover, the outcome data extracted for each of the included studies are presented in **Supplementary Table 3**.

### 3.3 Quality assessment

The Cochrane Risk of Bias (RoB 2) tool assessed 9 RCTs and found that three trials (12, 20, 27) showed low overall risk of bias. **Table 2** summarizes the risk of bias assessment for the included RCTs. The five trials (18, 19, 21, 23, 26) received some concerns as showed D2 (deviations from intended interventions) and D3 (missing outcome data) discrepancies. Meanwhile, the trial (16) received high risk of bias assessment because its open-label design created major D2 concerns. The NOS assessment of seven observational studies found that some studies achieved NOS scores between 8 and 9 which indicated low risk of bias. **Table 3** presents the summary of quality assessment fr the included observational cohort studies. The studies by Bayoumi et al. (13) and Davis et al. (15) received scores between 6 and 8 points which indicated moderate risk whilst Lapertosa et al. (17) received 7 points which showed moderate risk because of comparability and follow-up limitations.

**Table 2:** Assessment of risk of bias of the included RCTs.

Studies	D1	D2	D3	D4	D5	Overall risk of bias
20	Low	Low	Low	Low	Low	Low
23	Low	Some concerns	Some concerns	Low	Low	Some concerns
19	Low	Some concerns	Low	Some concerns	Low	Some concerns
18	Low	Low	Some concerns	Low	Low	Some concerns
26	Low	Some concerns	Some concerns	Low	Low	Some concerns
12	Low	Low	Low	Low	Low	Low
27	Low	Some concerns	Low	Low	Low	Some concerns
21	Low	Some concerns	Some concerns	Low	Some concerns	Some concerns
16	Low	High	Low	Low	Low	High

D1, randomization process; D2, deviations from intended interventions; D3, missing outcome data; D4, measurement of the outcome; D5, selection of the reported result.

**Table 3:** Quality assessment of the included observational cohorts.

Studies	Selection				Comparability		Outcome			Overall Quality	
	Representativeness of exposed cohort	Selection of non-exposed cohort	Ascertainment of exposure	Outcome not present	Comparability - design/analysis	Comparability - additional confounders controlled	Assessment of outcome	Follow-up long enough for outcome	Adequacy of follow-up	Total NOS score	Risk judgement
25	*	*	*	*	*	*	*	*	*	9	Low
13	*	*	*	*	*	*	*	*	0	8	Low
22	*	*	*	*	*	*	*	*	*	9	Low
24	*	*	*	*	*	*	*	*	*	9	Low
17	*	*	*	*	*	0	*	*	0	7	Moderate
15	*	0	*	*	*	0	*	*	0	6	Moderate
14	*	*	*	*	*	*	*	0	*	8	Low

NOS, NewCastle Ottawa scale.

* denotes one star (point) awarded on the corresponding Newcastle-Ottawa Scale (NOS) item, indicating that the criterion was met; "0" indicates that the criterion was not met. The total NOS score equals the sum of the stars awarded (maximum 9).

### 3.4 Results of meta-analysis

#### 3.4.1 Glyceamic profile

The meta-analysis of fasting glucose combined data from six studies (15, 18, 19, 21, 23, 26). The study results showed a MD = -0.02 mmol/L (95% CI: -1.52 to 1.49; z = -0.02; p = 0.98), which supported that there was no significant net effect amongst the studies (**Figure 2a**). The study results showed substantial heterogeneity through I² = 80.7%, tau² = 3.10, and Q = 25.86 with df = 5 and p < 0.0001. The study showed a large positive difference through Hawkins et al. with MD of +4.60 mmol/L (95% CI: 1.59–7.61) for a lifestyle intervention study that used glucose measurement after meals with an extremely high baseline standard deviation (19). The study evaluated pre-pregnancy weight loss before a GDM pregnancy, which showed a reduction of -3.60 mmol/L (95% CI: -5.81 to -1.39) (21). The findings suggest that fasting glucose outcomes between studies show their major differences through variations in measurement units and timing and the type of intervention used.

**Figure 2:**
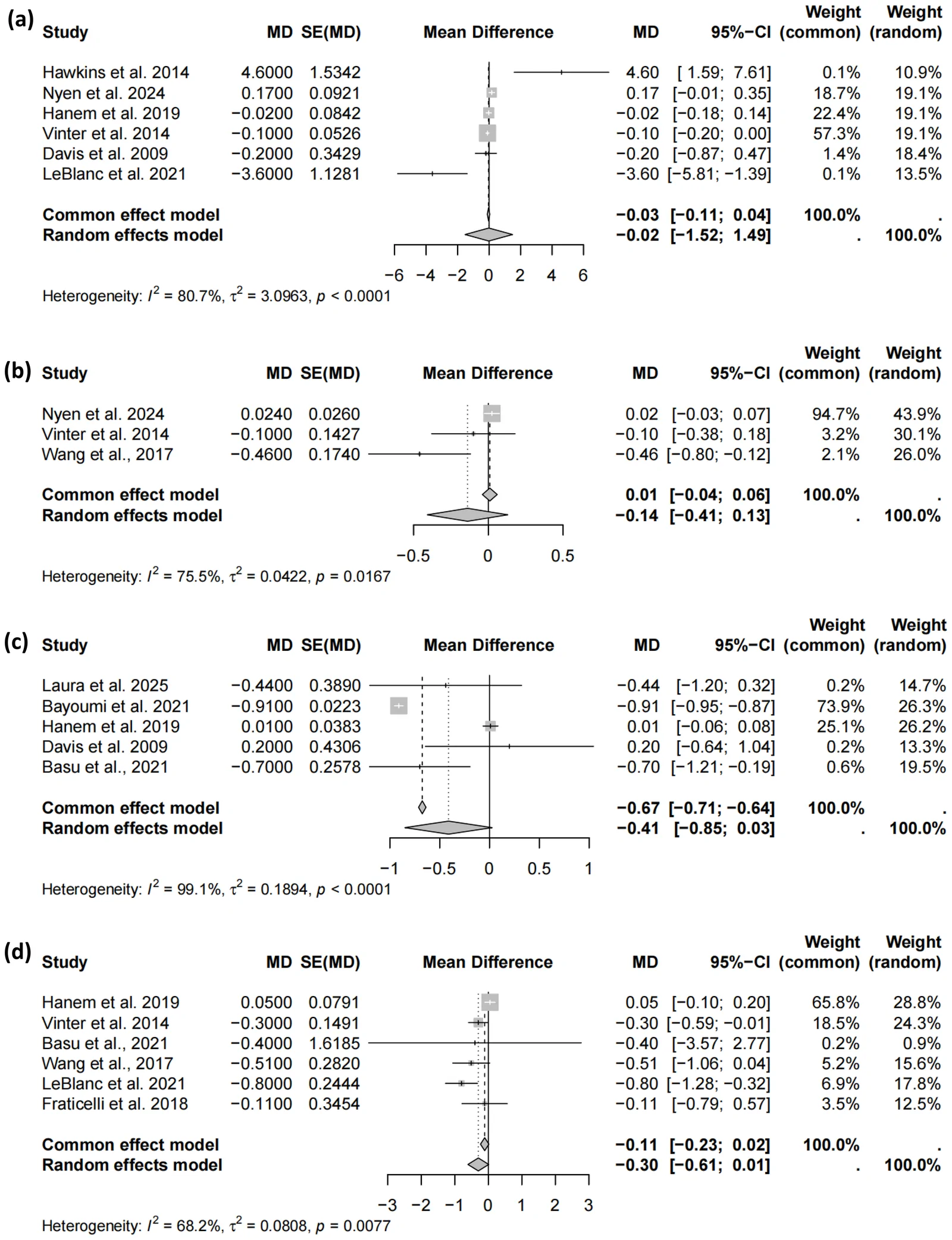
Mean difference analysis of glycaemic profile **(a)** fasting glucose, **(b)** 2hOGTT, **(c)** HbA1c, and **(d)** HOMA-IR. MD with 95% CI was illustrated in the forest plot using a random-effects model.

##### 3.4.1.1 2-hour post-OGTT glucose

Three studies reported 2-hour OGTT values (23, 26, 27). The pooled random-effects estimate resulted in a mean difference of MD = -0.14 mmol/L (95% CI: -0.41 to 0.13; z = -1.01; p = 0.31), which did not reach statistical significance (**Figure 2b**). The study showed moderate to high heterogeneity through I² = 75.5% and Q = 8.18 with two degrees of freedom and p = 0.017. The study that Wang et al. conducted in Chinese women through an exercise RCT showed the highest effect with an MD of -0.46 mmol/L (95% CI: -0.80 to -0.12) whilst Nyen et al. showed a negligible effect with an MD of 0.024 (95% CI: -0.027 to 0.075) (23).

##### 3.4.1.2 HbA1c

The studies evaluated five studies which measured HbA1c results through their research. The random-effects model produced a pooled MD value of -0.41% with a 95% CI range from -0.85 to 0.03 and a z value of -1.84 which produced a p value of 0.07 (**Figure 2c**). The study results showed extreme between-study variation which produced an I² value of 99.1% and a Q value of 436.23 with 4 degrees of freedom and a p value less than 0.0001 and a tau² value of 0.19. The results from the GDM observational study conducted by Bayoumi et al. with 5043 participants showed a 0.91% decrease which produced a 95% CI range from -0.95 to -0.87. The high I² value requires careful study results evaluation because active GDM treatment resulted in HbA1c reduction which indicated treatment effects but did not show actual reduction of metabolic risk for GDM-based offspring.

##### 3.4.1.3 HOMA-IR

The research included HOMA-IR data from six studies. The random-effects model produced a pooled estimate which showed MD value of -0.30 with 95% CI range from -0.61 to 0.01 and a z value of -1.88 which resulted in a p value of 0.060 (**Figure 2d**). The study results showed moderate heterogeneity with I² value of 68.2% and Q value of 15.73 at 5 degrees of freedom and a p value of 0.008. Vinter et al. (obese women) reported lifestyle RCT results which showed HOMA-IR value decreased with statistical significance through an MD value of -0.30 from 95% CI range of -0.59 to -0.008 and a p value of 0.044). LeBlanc et al. showed HOMA-IR results which reached statistical significance with an MD of -0.80 and a 95% confidence interval which ranged from -1.28 to -0.32 and a p value of 0.001. Hanem et al. (metformin RCT) showed non-significant positive trend for offspring HOMA-IR results based on their intervention method which produced a 0.05 value (18).

#### 3.4.2 Adiposity outcomes

##### 3.4.2.1 BMI and overweight/obesity

The studies observed elevated offspring BMI as the most common result across five investigations that measured BMI and obesity-related outcomes. The observational studies contained the most valuable information because they studied two groups which found to have different rates of overweight in adults whom GDM diet-treated pregnancies produced compared to adults whose parents had normoglycaemic pregnancies (OR = 2.09, 95% CI: 1.25–3.50) and found that the first group had higher rates of metabolic syndrome (MD = 5.35, 95% CI: 2.31–12.42). The GDM-exposed offspring showed higher BMI values (MD = 2.01, 95% CI: 1.49–2.71), as shown in **Figure 3**. Hanem et al. conducted RCTs which showed that offspring BMI values showed greater weight gain when participants received placebo treatment instead of metformin treatment (MD = +1.02 kg/m², 95% CI: 0.07–1.97) which raised doubts about metformin use during pregnancy because it might lead to increased body fat in children (18). Pregnant women who experienced GDM showed higher weight gain during pregnancy reported a rate ratio of 1.25 (95% CI: 1.10–1.42).

**Figure 3:**
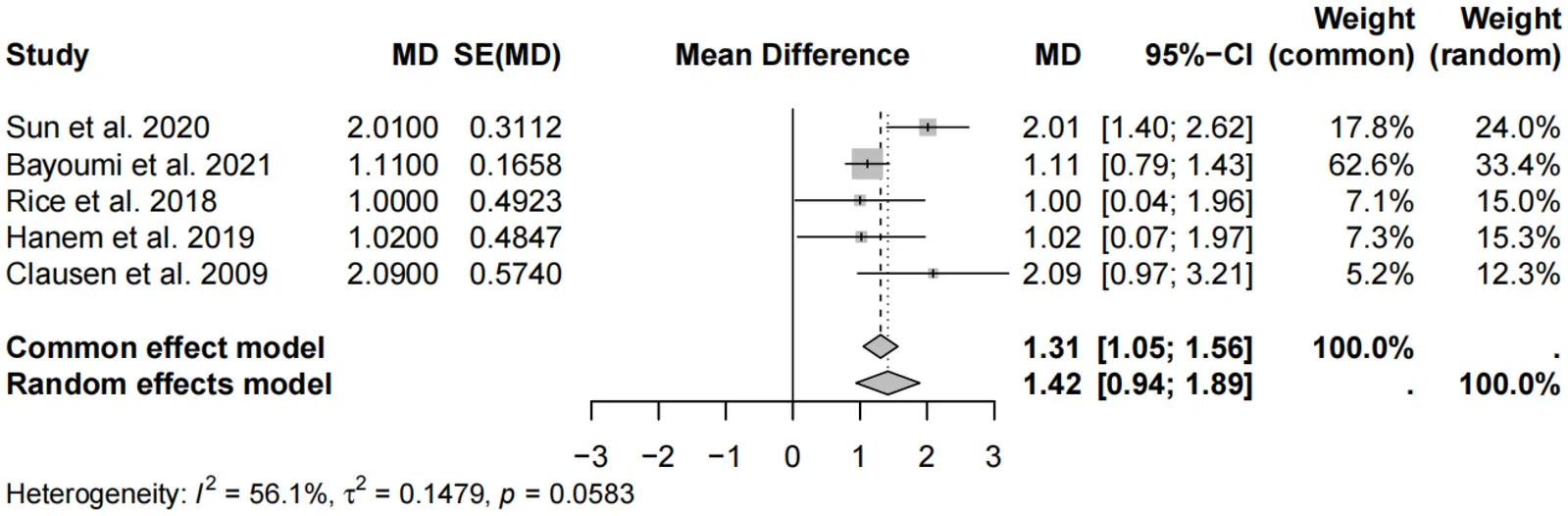
BMI (Strand A: Exposure) values evaluated across the included studies reported MD of 1.42 with 95% CI between 0.94 and 1.89, represented through forest plot using a random-effects model.

##### 3.4.2.2 Waist circumference and body fat percentage

Rice et al. and Hanem et al. provided three data points which researchers used to measure adiposity markers (**Figure 4**) (18). The two studies showed that GDM-exposed offspring had higher waist circumference measurements which reached a pooled MD range of approximately +2.27 to +3.01 cm although most tests showed zero results in their confidence intervals. The study results showed that Hanem et al. metformin-treated offspring reached higher body fat percentages than the placebo group (MD = +3.04% 95% CI -0.6 to 6.7) (18).

**Figure 4:**
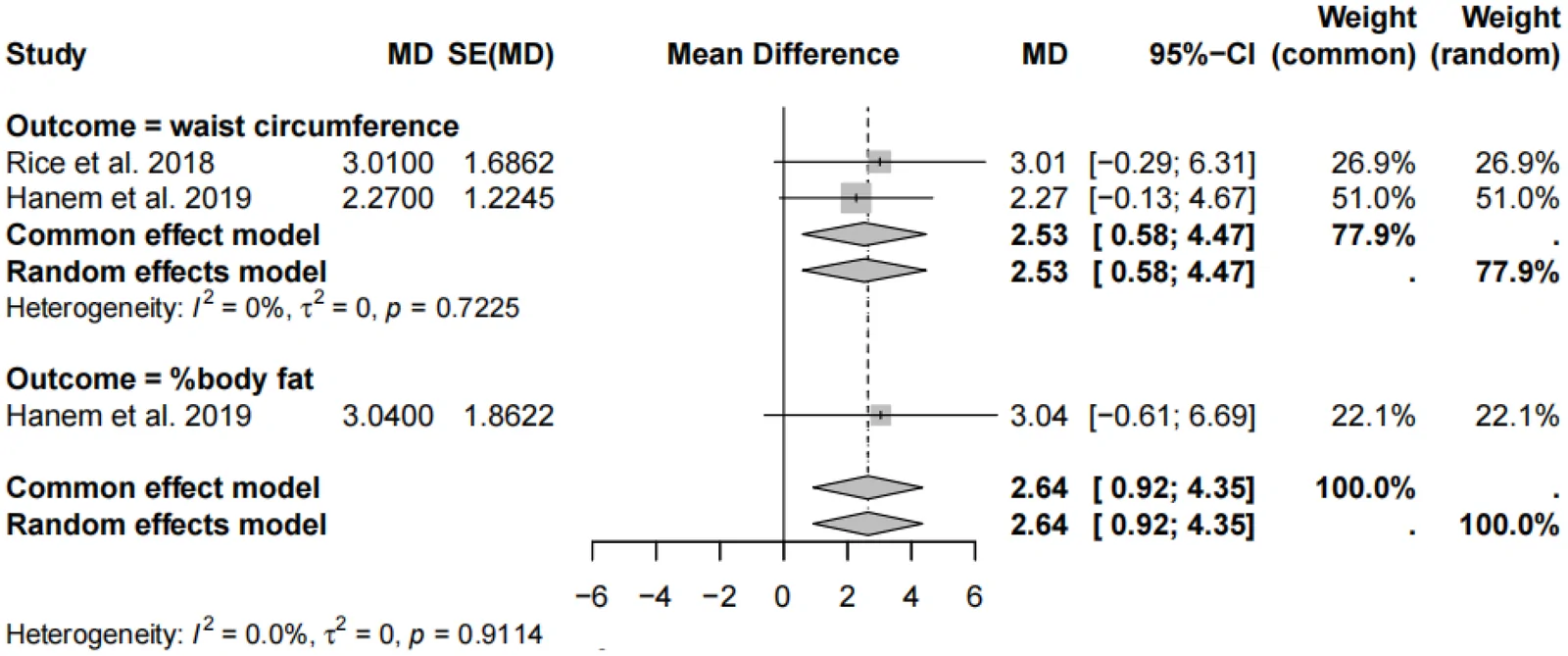
Waist circumference and % body fat presented MD with 95% CIs and heterogeneity within the defined population with GDM across the included studies.

#### 3.4.3 Blood pressure outcomes

##### 3.4.3.1 Systolic and diastolic blood pressure

The blood pressure assessments for this study included results from four research groups which operated two independent RCTs (18, 20), as shown in **Figure 5**. The combined MD for SBP approached zero which Kusinski et al. reported as -0.96 mmHg with a 95% CI of -3.50 to 1.58 and Hanem et al. reported as +0.95 mmHg with a 95% CI of -1.87 to 3.76 (18, 20). Diastolic blood pressure (DBP) measurements showed results that were not statistically significant. Clausen et al. showed that GDM offspring experienced higher rates of hypertension which affected 37% of GDM offspring and 24% of non-GDM offspring for SBP elevation and 4% of GDM offspring and 2% of non-GDM offspring for DBP elevation. Rice et al. discovered that GDM-exposed 7-year-old children exhibited increasing blood pressure levels, MD approximately 1 mmHg (95% CI: 0.03 to 1.96).

**Figure 5:**
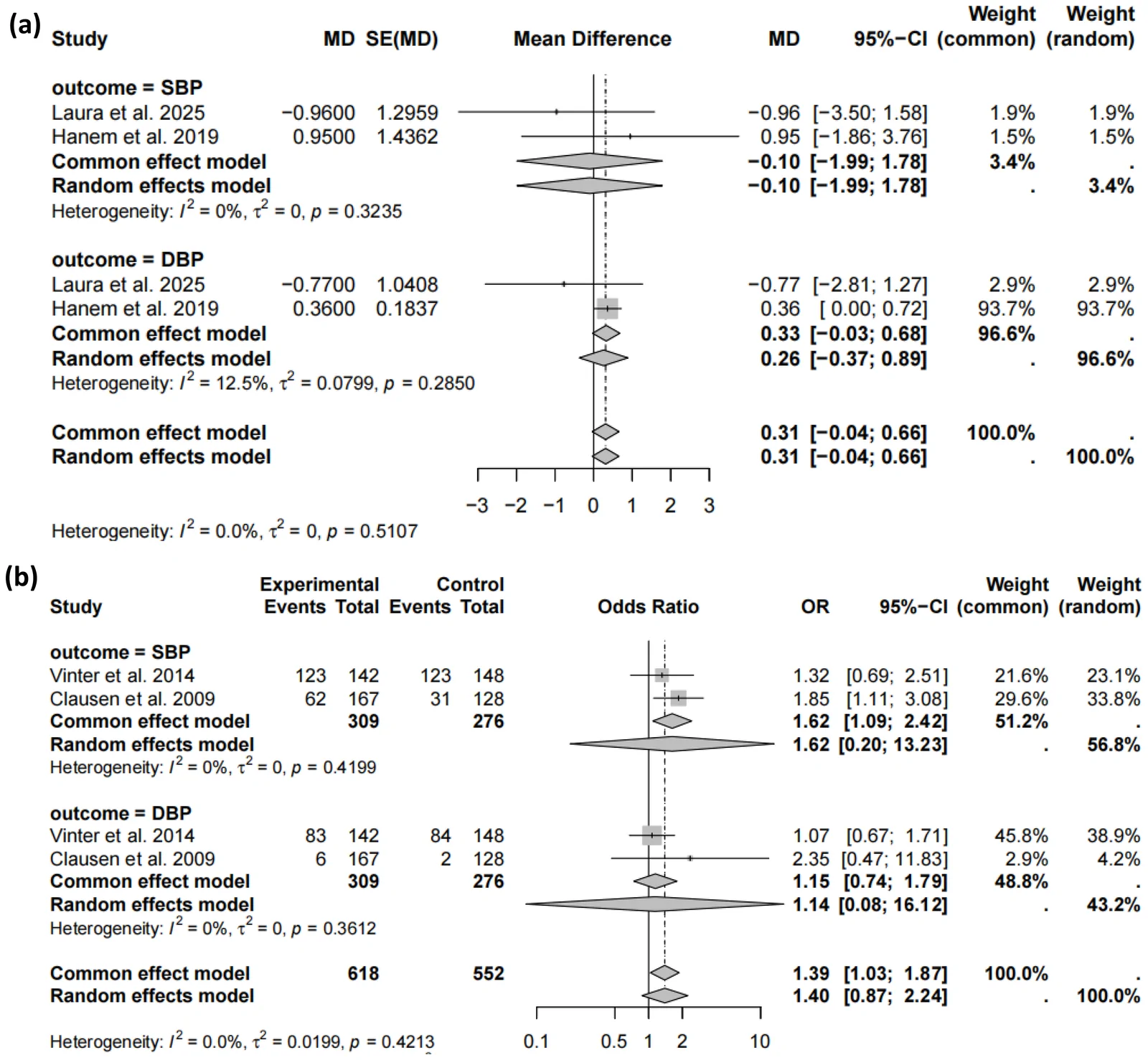
Hypertension-related outcome data was divided into two analysis **(a)** MD with 95% CI and **(b)** OR with 95% CI and heterogeneity. Where both of the analyses reported zero heterogeneity within SBP and DBP within experimental and control groups.

#### 3.4.4 Lipid profile

##### 3.4.4.1 Total cholesterol

The total cholesterol analysis included data from four different studies (**Figure 6**). The random-effects estimate showed a pooled result of MD = +0.07 mmol/L with a 95% confidence interval of -0.07 to 0.21 which resulted in a z-value of 0.93 and a p-value of 0.35. The study found no significant heterogeneity because I² equalled 0.0% and the Q statistical test produced results of 2.60 with three degrees of freedom and a p-value of 0.46. The study shows that GDM management interventions do not produce substantial changes in total cholesterol levels amongst offspring because of their null finding which maintains uniformity across their results.

**Figure 6:**
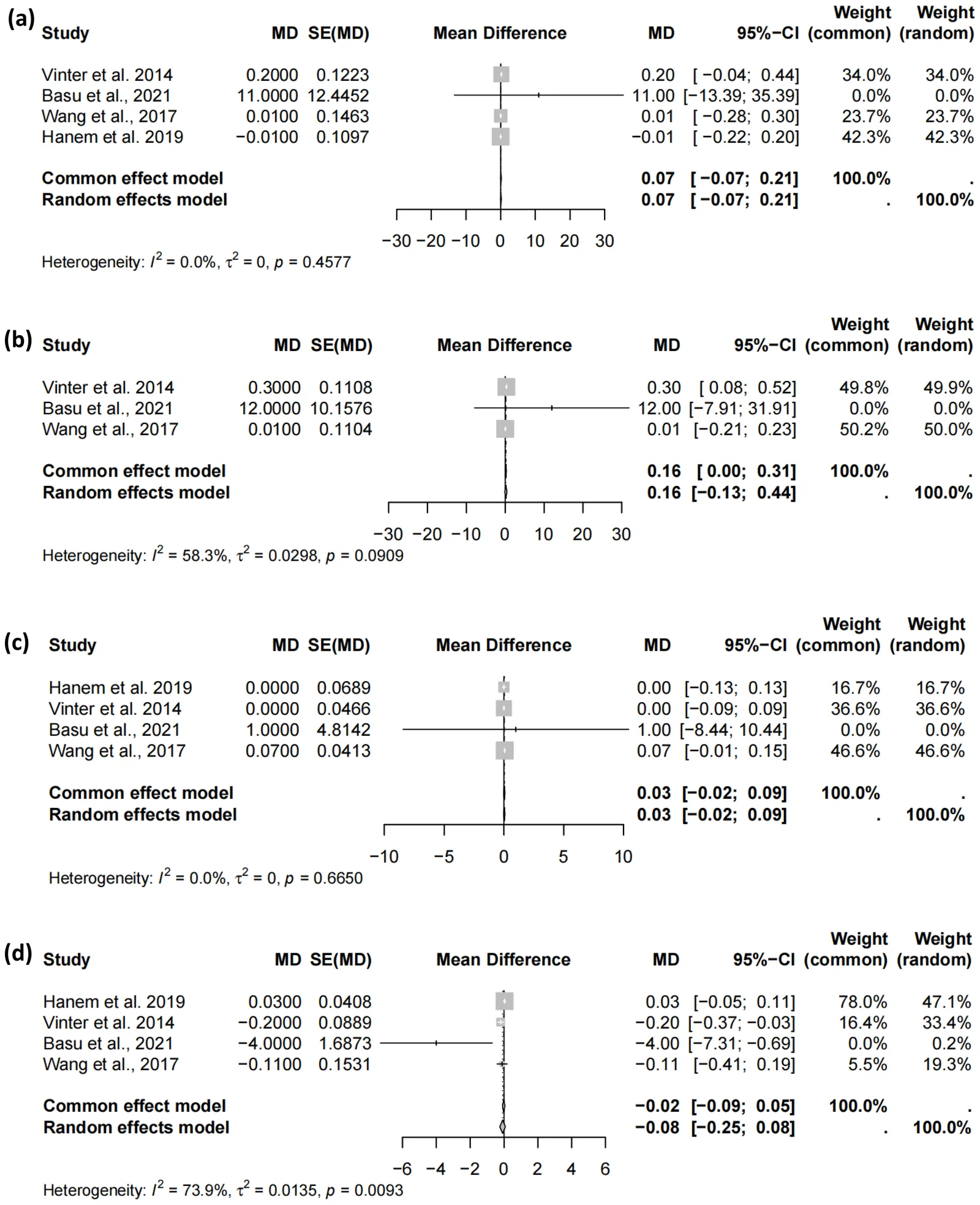
Mean difference analysis of lipid profile **(a)** total cholesterol, **(b)** LDL-C, **(c)** HDL-C, and **(d)** triglycerides. MD with 95% CI and heterogeneity was illustrated in the forest plot using a random-effects model.

##### 3.4.4.2 LDL-cholesterol

Three studies reported LDL-C outcomes. The random-effects model showed non-significant pooled effect (MD = +0.16, 95% CI: -0.13-0.44, p-value = 0.28) and I² of 58.3%. (26) produced the most exact measurement which verified that LDL-C levels increased significantly in the lifestyle intervention group.

##### 3.4.4.3 HDL-cholesterol

Four studies provided evidence for HDL-C assessment through their analysis. The random-effects model produced a pooled mean difference of 0.033 mmol/L which showed no statistical indices of variation between studies because I² showed 0.0% and Q tested at 1.58 with three degrees of freedom whilst showing p value of 0.67. GDM management approaches and GDM exposure do not produce significant effects on HDL-C levels.

##### 3.4.4.4 Triglycerides

The random-effects model produced a pooled estimate which showed MD value of -0.084 mmol/L with a 95% confidence interval from -0.25 to 0.08 and z score of -0.99 and p value of 0.32. Basu et al. found that blueberry and fibre supplementation produced a substantial decrease in triglyceride levels which showed an MD value of -4.00 mmol/L but researchers could not combine results because of the wide confidence intervals and extreme baseline values which showed a mean of 9.5 mmol/L in a population that showed hypertriglyceridaemia tendencies.

### 3.5 Publication bias assessment

The analysis used Egger’s linear regression test to examine funnel plot asymmetry of the glucose pool which contained 20 effect estimates and the lipid pool which contained 15 effect estimates (**Figure 7**). The combined glucose pool test produced t = 0.82 (df = 18; p = 0.43) with a bias estimate of 1.79 (SE = 2.20) which showed no evidence of publication bias. The test for the combined lipid pool produced test results of t = -0.08 (df = 13; p = 0.94) which showed that funnel asymmetry did not exist because the bias estimate was -0.04 (SE = 0.56). The visual inspection of funnel plots supported the results which had been obtained through statistical methods. The results indicate that the analysed literature does not show significant impact from small-study and publication bias in the examined research domains.

**Figure 7:**
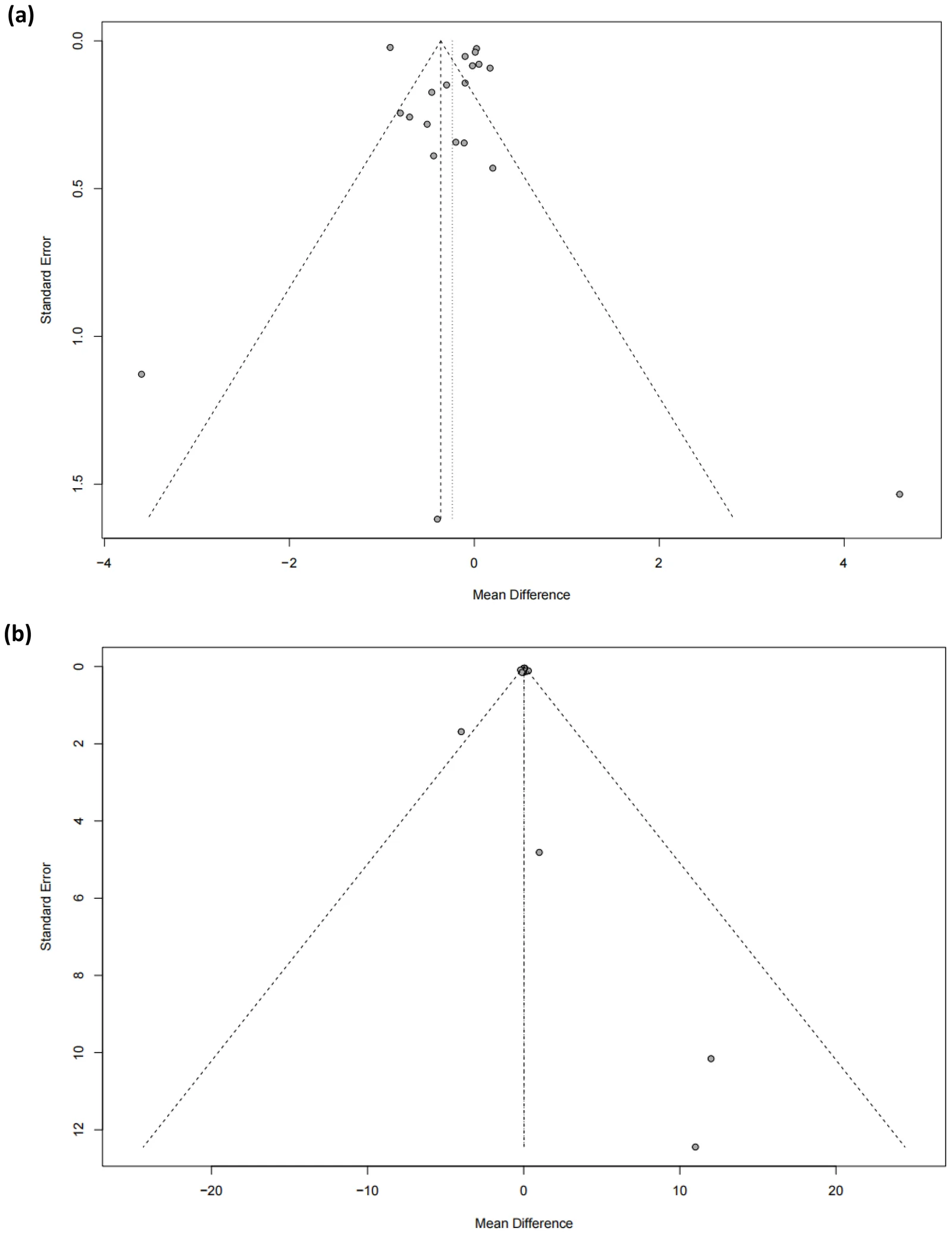
Publication bias assessment **(a)** glycaemic and **(b)** lipid profile reported within the included studies.

### 3.6 Subgroup and sensitivity analyses

Subgroup analyses were performed to study glycaemic and lipid outcomes based on different intervention types. The metformin subgroup (18) indicated results which differed from the dietary/lifestyle subgroup specifically regarding adiposity and blood pressure measurements which needed independent evaluation during clinical practice, as shown in **Figures 8**, **9**. Furthermore, the sensitivity analysis for both glycaemic and lipid outcomes were also shown in **Figure 10**.

**Figure 8:**
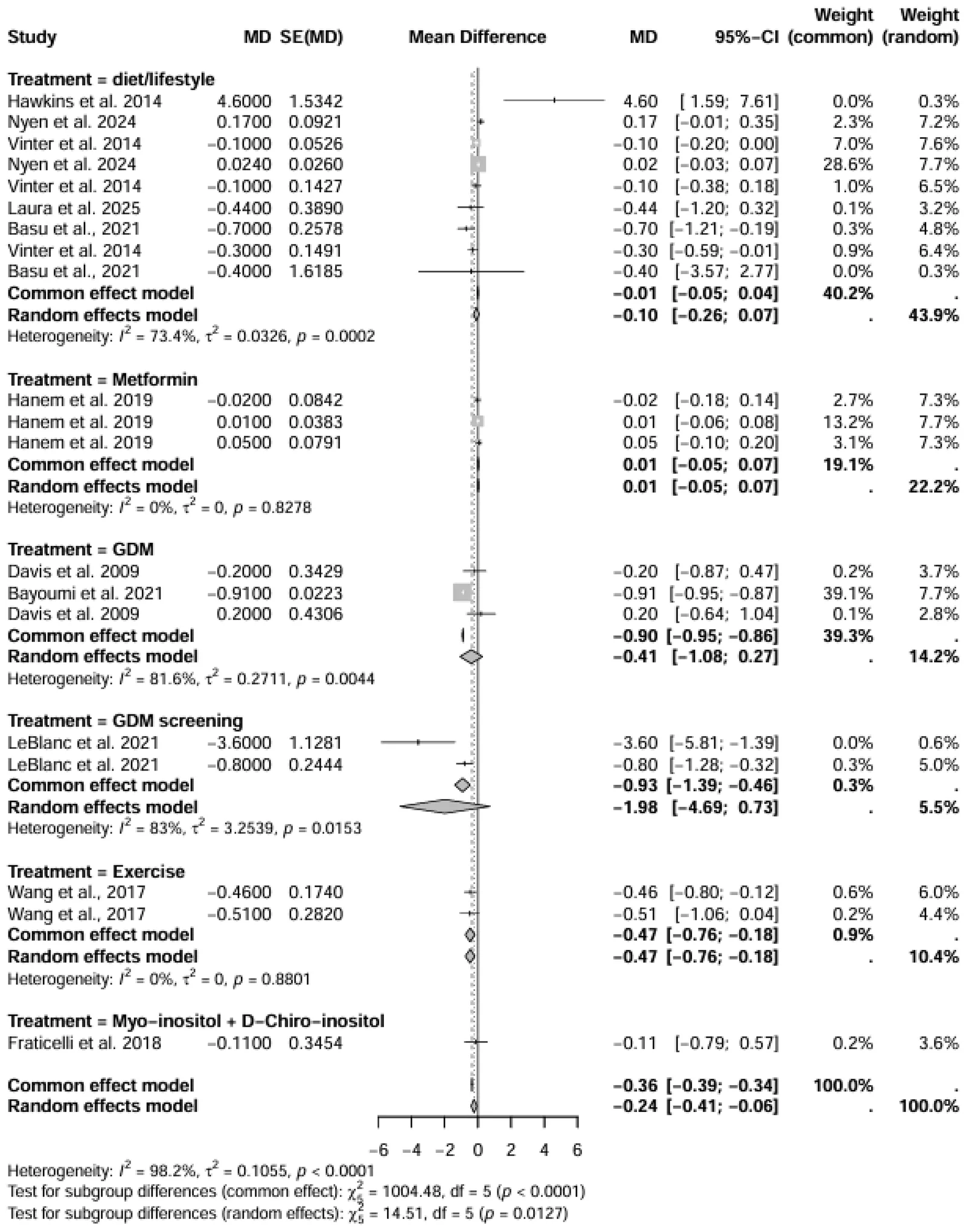
Subgroup analysis of glycaemic profile based on treatment groups.

**Figure 9:**
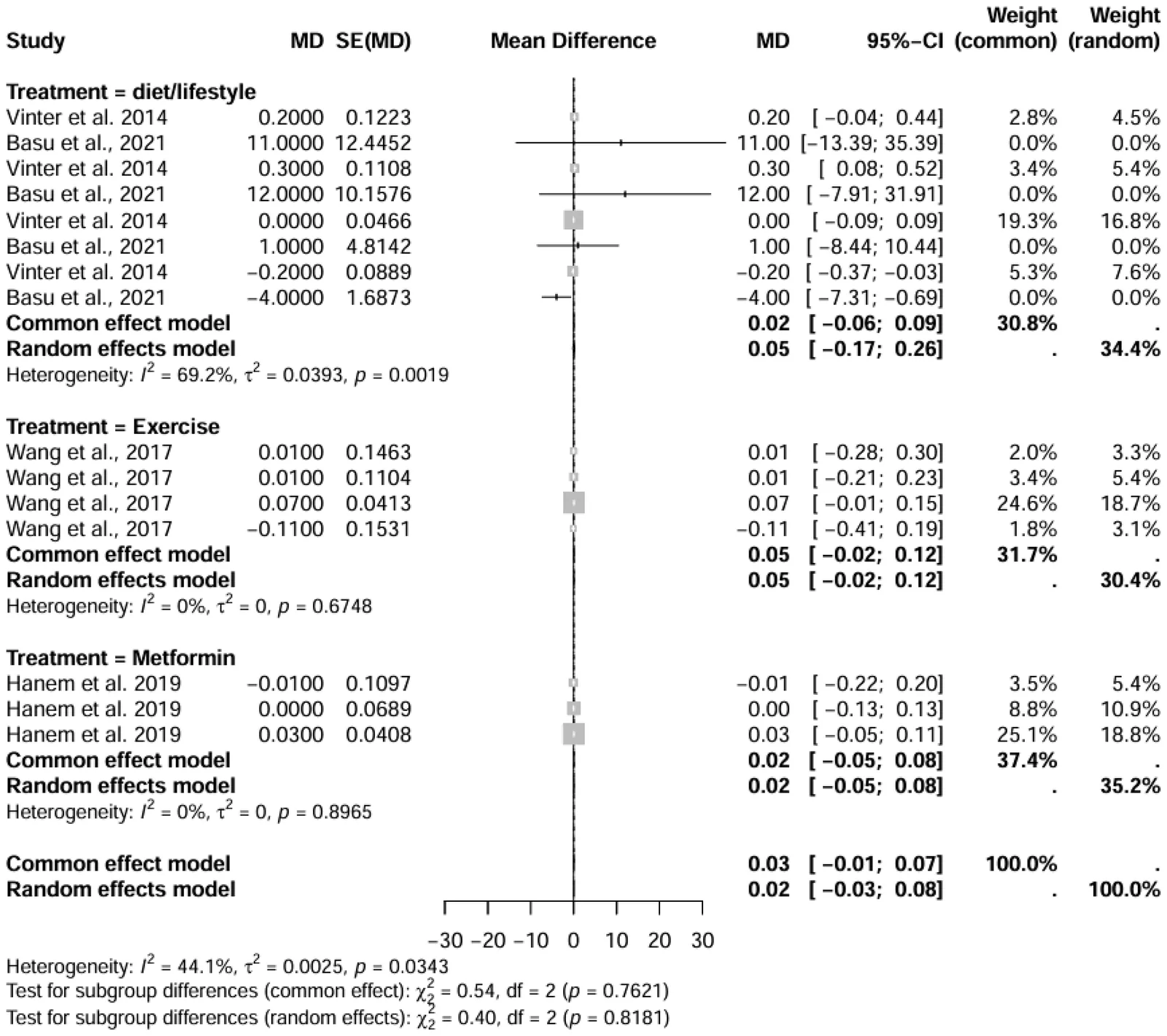
Subgroup analysis of lipid profile presented across the studies based on different treatment groups.

**Figure 10:**
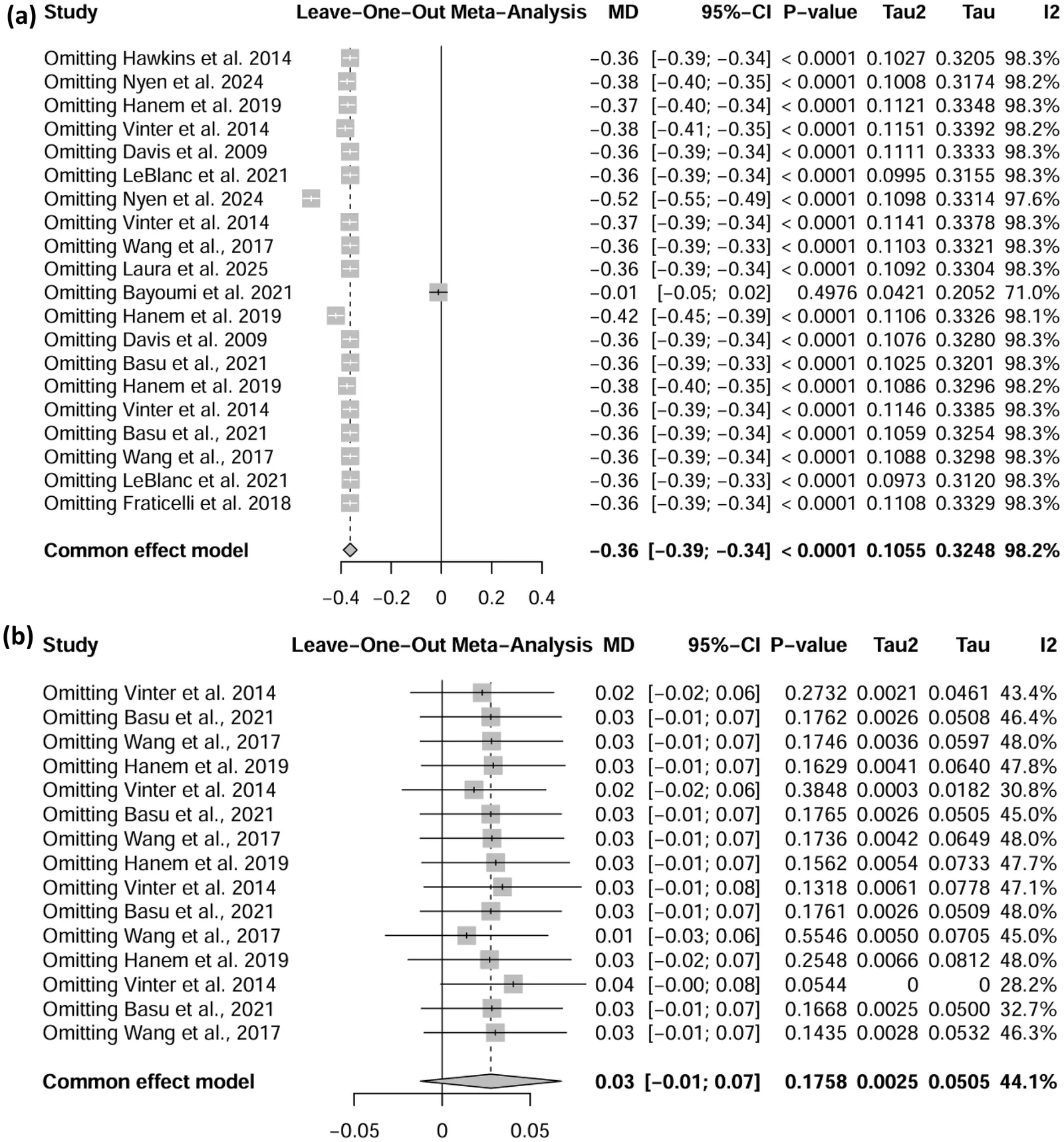
Sensitivity analyses for the **(a)** glycaemic and **(b)** lipid parameters reported amongst the included studies.

## 4 Discussion

The systematic review and meta-analysis which includes 16 studies from 11 different countries that were published between 2009 and 2025 provides complete quantitative results about how *in-utero* GDM exposure affects metabolic function across different health outcomes. The principal finding suggests that GDM-exposed offspring develop a specific risk profile which shows increased body fat and develops into dysglycaemia under testing conditions and shows a pattern of decreasing insulin sensitivity whilst their lipid levels and blood pressure state mixed effects which have little clinical significance through their diabetes management treatments. The study results are essential for clinical practice because they suggest that GDM functions as an intergenerational condition which produces fetal metabolic programming effects that last beyond neonatal development. Therefore, this review present evidence from observational studies that evaluates associations between prenatal GDM exposure and offspring metabolic outcomes, and randomized trials that test maternal interventions during pregnancy. Both of these evidence streams were interconnected but in a complementary way, so should not be interpreted as addressing the same causal questions.

The combined analysis of glycaemic results shows two distinct outcomes which need specific contextual information to be understood properly. The fasting glucose results showed no significant pooled effect with an MD of -0.016 mmol/L and a p value of 0.98 whilst the included studies had 80.7% I² which showed their results had important population-based and intervention-based and measurement-based differences. The LeBlanc et al. found that pre-pregnancy weight loss as a GDM prevention method created a major weight-adjusted effect which showed that decreased hyperglycaemia duration improved offspring fasting glucose levels according to the DOHaD model (2). The research from Hawkins et al. shows through their small RCT study that their results present higher average fasting glucose rates for the intervention group (19). The active GDM management shows a trend of decreasing HbA1c levels which medically makes sense but the results from Bayoumi et al. observational study show greater statistical impact. Different studies face a major problem because they assess HbA1c levels at various developmental stages which include neonates and children and adults. The future meta-analyses should use age-specific measurement time points to create separate groups for their offspring outcomes. The HOMA-IR results provide the most useful information for clinical practice. The GDM management interventions show a trend towards reduced insulin resistance because women who achieve glycaemic control during their pregnancies will decrease the intergenerational insulin resistance which doctors believe happens from fetal hyperinsulinism and beta-cell overstimulation. The results from Vinter et al. and LeBlanc et al. show significant effects which support this interpretation. The metformin arm of Hanem et al. shows an unexpected HOMA-IR effect that shows positive results because metformin has multiple effects which include its ability to activate AMPK and control mitochondrial activity (18). The PregMet trial design shows residual confounding because it allows indication-based treatment assignment.

The observational studies which directly study whether GDM exposure increases offspring adiposity provide the strongest evidence on this subject. Clausen et al. used a prospective design to track 293 adult children from GDM diet-based treatment and 128 control children until they reached the average age of 21.6 years which showed that GDM diet-based treatment increased the risk of overweight (OR = 2.09, 95% CI: 1.25–3.50) and metabolic syndrome (OR = 5.35, 95% CI: 2.31–12.42) despite controlling for maternal pre-pregnancy body mass index and other confounding variables. Sun et al. found that GDM increased the risk of negative outcomes for mothers and newborns after they studied a large Chinese multicentre research population. The observational studies produced results which showed large effects which are consistent with the EPOCH cohort findings from Dabelea et al. and the HAPO Follow-Up Study results from Lowe et al. thus supporting our findings with strong external validity (7, 28).

Hanem et al. shows that metformin exposure during pregnancy leads to increased offspring BMI which showed a measurement of MD = +1.02 kg/m² at 5–10 years of age (18). The high-quality PedMet follow-up of the PregMet RCT produced results which identify a crucial pharmacological safety signal that the MiTy Kids trial and the Northern Hypoglycaemia follow-up study have confirmed. When metformin passes through the placental barrier to enter the developing foetus at therapeutic dosage levels medical research shows (14) that this drug can change fetal fat development through AMPK-mTOR pathways which result in increased fat cell growth and improved fat storage capability. The scientific mechanism should undergo examination through research that uses specialized research setups to test its validity.

The study found no important combined effects on total cholesterol and HDL-C and triglyceride levels which showed minimal to no variations between study sites. The three to four studies that participated in this research used mainly brief RCTs which created insufficient statistical strength to identify small yet significant changes in lipid levels. Clausen et al. showed that young adult offspring of GDM mothers experienced much higher dyslipidaemia rates (elevated total cholesterol: 37% vs. 13%; elevated triglycerides: 16% vs. 5%) which RCT data from brief antenatal interventions used in meta-analyses could not show. The common-effect model showed borderline significant LDL-C elevation (MD = +0.16 mmol/L; p = 0.047) which needs further research because of the connection between LDL-C levels and cardiovascular disease development over time.

The study results on blood pressure were restricted because only a few studies met eligibility requirements and the RCTs included in the research used short-term follow-up periods. The evidence from Rice et al. (children at age 7) and Clausen et al. (young adults) provides more substantial information which reveals that GDM offspring show a small but clinically significant increase in SBP. The evidence supports the theory which states that intrauterine hyperglycaemia disrupts both nephrogenesis and renin-angiotensin system development. The field requires larger longitudinal research which should systematically assess blood pressure levels in GDM offspring.

The glycaemic outcome analyses displayed substantial heterogeneity which persisted throughout all tests (I² = 68–99%) and multiple lipid tests (triglycerides I² = 74%). Multiple sources of clinical heterogeneity plausibly contribute. First, GDM diagnostic criteria varied markedly: studies using the more sensitive IADPSG criteria include a broader spectrum of maternal hyperglycaemia severity than those using the more restrictive Carpenter-Coustan or NDDG criteria, and offspring risk may vary accordingly. The GDM management procedures include diet and lifestyle changes and drug treatment which produce multiple different exposure patterns that affect how children develop. The measurement of metabolic risk markers shows major development changes from the measurement of outcomes in neonates to young adults (14). The 11 countries included in the study show ethnic and environmental diversity which results in different metabolic outcomes because of variations in genetic makeup and dietary habits and exercise patterns and economic conditions. The heterogeneous sources present challenges for meta-analytic pooling because they show how GDM exists as a worldwide heterogeneous medical condition. The random-effects model serves as the correct analytical method to use because researchers should interpret the combined results through their associated prediction intervals which show all possible outcome effects.

Persistent high heterogeneity (I² 68–99%) shows three residual sources: (i) the offspring age when the outcome is assessed, which goes from neonatal up to young adulthood across different studies; (ii) the GDM diagnostic thresholds kept changing over the 2000–2026 search window (IADPSG vs. Carpenter-Coustan vs. NDDG), so the severity range of maternal hyperglycaemia being captured shifts; and (iii) maternal pre-pregnancy BMI, which is reported rather inconsistently across cohorts. In other words, these points are treated more as limitations rather than resolve statistically, and prediction intervals are shown alongside pooled estimates whenever feasible.

The findings present multiple specific points which organizations can implement. The results emphasize that GDM pregnancies need optimal glycaemic control because it protects neonatal health whilst preventing future metabolic disorders in the child. The recommendation receives support from research results which show decrease in HOMA-IR (21, 26). The GDM-related BMI outcomes show lower results in well-managed cohorts. The intrauterine metformin exposure study shows a pharmacological safety signal which needs studying yet should impact clinical decisions about GDM women who need medication. The GDM offspring group represents a high-risk population which needs metabolic surveillance from childhood through adolescence. They need regular screening for obesity and impaired glucose tolerance and dyslipidaemia at intervals which match the severity of their intrauterine exposure. The research shows that GDM exposure leads to five times greater risk for developing adult metabolic syndrome according to their study results (OR = 5.35; 14). Pre-conception interventions (weight management, lifestyle modification) that prevent or reduce the severity of GDM may represent high-value intergenerational health investments.

The review suggests significant strengths through its extensive coverage of multiple metabolic domains and its use of validated risk of bias assessment tools and its application of Egger’s test for publication bias evaluation. The inclusion of both RCTs and observational studies allows triangulation of evidence from intervention contexts and real-world epidemiological settings. The study includes certain limitations. A major limitation of this review is that it includes both observational and randomized studies, which address related but distinct clinical questions. Even so, these studies were put together based on the outcome, but the conclusions were interpreted separately, so as to not inadvertently mix findings that are linked with exposure, and the true effects that come from an intervention. The statistical power of the study decreases because there are only three to six studies available to examine each outcome domain. The research results should not be considered universal GDM effects because the study results contained significant clinical and methodological differences which the researchers managed through modelling. Most RCTs designed their studies to evaluate metabolic outcomes in offspring as secondary or exploratory endpoints which created measurement challenges and reporting difficulties. The brief follow-up period of RCTs which examine participants only until their neonatal stage or their early childhood stage prevents researchers from assessing long-term effects which develop during adolescence and adulthood when GDM exposure most clearly produces metabolic effects. The studies showed different results because researchers applied postnatal confounder adjustments (breastfeeding, postnatal diet, physical activity, parental BMI at follow-up) in various ways which made it hard to determine how much intrauterine factors contributed to postnatal development.

The review identifies several priority areas for future research which need to be investigated. Dedicated prospective longitudinal cohort studies of GDM offspring with standardised metabolic phenotyping at pre-specified developmental time points (birth, 5 years, 10 years, 18 years, 30 years) are needed. Researchers should define GDM metabolic outcomes through pre-registration as primary study endpoints and they should apply standardised measurement methods which enable tracking participants over an extended period. The development and validation of a GDM offspring metabolic risk score, which combines exposure severity assessment with treatment method evaluation and primary postnatal factors, would enable precise patient risk evaluation. Mechanistic studies which investigate how GDM offspring epigenetic changes (DNA methylation, histone modification) in cord blood develop throughout their lives, will provide scientific support for the DOHaD framework in this research area.

## 5 Conclusion

The systematic review and meta-analysis identifies a consistent association between *in-utero* GDM exposure and adverse offspring metabolic patterns in observational cohorts. Whilst, a statistically supported benefit of active antenatal glycaemic management on offspring insulin resistance was observed in clinical trials. The evidence shows highest reliability for increased body fat together with overweight risk because observational data shows that young adults who had GDM exposure have more than twice the odds of becoming overweight and they experience a fivefold greater risk of developing metabolic syndrome compared to people with normal blood sugar levels. The active management of GDM shows a clinically significant improvement in HOMA-IR and other glycaemic outcomes which establishes that intrauterine treatments can decrease metabolic programming effects on offspring. The available evidence shows that both lipid abnormalities and blood pressure increases exist according to observational studies but RCT analyses need more proof because of study design restrictions which show results but not actual impact.

The existing high variation in glycaemic results necessitates GDM-offspring research to use standardised diagnostic criteria together with consistent outcome assessment methods and comprehensive long-term patient monitoring. The safety signal for metformin exposure in pregnancy requires further research through a prospective evaluation study. GDM constitutes a medical condition which creates metabolic health problems for future generations so healthcare professionals and government officials need to handle GDM through improved blood sugar control during pregnancy together with planned metabolic health monitoring for children born to mothers with GDM. The implementation of these evidence-based measures creates a vital chance to break the cycle of metabolic disorders which moves from one generation to another.
