# Supplementary material for: Long-term metabolic consequences of in-utero gestational diabetes exposure and maternal treatment interventions: a systematic review and meta-analysis
Source: Front Endocrinol (Lausanne). 2026 Sep 16;17:1904624. doi: 10.3389/fendo.2026.1904624 (PMC13623741; doi:10.3389/fendo.2026.1904624)
Supplement: Supplementary file 1 [file Table1.docx]

**Supplementary Materials**

**Table S1:** PubMed search query.

| Search number | Query | Filters | Results | Time | Date |
| --- | --- | --- | --- | --- | --- |
| 24 | #3 AND #7 AND #17 | Abstract, Full text, Clinical Study, Comparative Study, Observational Study, English, Humans, from 2000 - 2026 | 722 | 4:06:16 | 4/25/2026 |
| 23 | #3 AND #7 AND #17 | Abstract, Full text, Clinical Study, Comparative Study, Observational Study, English, from 2000 - 2026 | 726 | 4:06:10 | 4/25/2026 |
| 22 | #3 AND #7 AND #17 | Abstract, Full text, Clinical Study, Comparative Study, Observational Study, from 2000 - 2026 | 740 | 4:06:03 | 4/25/2026 |
| 21 | #3 AND #7 AND #17 | Abstract, Full text, from 2000 - 2026 | 5,451 | 4:05:20 | 4/25/2026 |
| 20 | #3 AND #7 AND #17 | Abstract, from 2000 - 2026 | 5,669 | 4:05:15 | 4/25/2026 |
| 19 | #3 AND #7 AND #17 | from 2000 - 2026 | 5,726 | 4:05:09 | 4/25/2026 |
| 18 | #3 AND #7 AND #17 |  | 5,946 | 4:04:56 | 4/25/2026 |
| 17 | #8 OR #9 OR #10 OR #11 OR #12 OR #13 OR #14 OR #15 OR #16 |  | 1,554,663 | 3:36:45 | 4/25/2026 |
| 16 | obesity[tiab] OR overweight[tiab] OR adiposity[tiab] OR body mass index[tiab] OR diabetes mellitus, type 2[tiab] OR insulin resistance[tiab] OR hypertension[tiab] OR dyslipidemias[tiab] OR metabolic syndrome[tiab] OR lipid disorders[tiab] | | 1,190,246 | 3:36:00 | 4/25/2026 |
| 15 | "Metabolic Syndrome"[Mesh] |  | 43,168 | 3:33:23 | 4/25/2026 |
| 14 | "Dyslipidemias"[Mesh] |  | 92,050 | 3:33:10 | 4/25/2026 |
| 13 | "Insulin Resistance"[Mesh] |  | 111,342 | 3:32:55 | 4/25/2026 |
| 12 | "Hypertension"[Mesh] |  | 338,238 | 3:32:27 | 4/25/2026 |
| 11 | "Diabetes Mellitus, Type 2"[Mesh] |  | 198,855 | 3:32:15 | 4/25/2026 |
| 10 | "Body Mass Index"[Mesh] |  | 167,000 | 3:32:07 | 4/25/2026 |
| 9 | "Overweight"[Mesh] |  | 306,583 | 3:31:52 | 4/25/2026 |
| 8 | "Obesity"[Mesh] |  | 294,351 | 3:31:42 | 4/25/2026 |
| 7 | #4 OR #5 OR #6 |  | 4,414,424 | 3:28:45 | 4/25/2026 |
| 6 | offspring[tiab] OR children[tiab] OR child[tiab] OR infant[tiab] OR adolescent[MeSH] OR young adult[MeSH] | | 4,414,424 | 3:28:33 | 4/25/2026 |
| 5 | "Young Adult"[Mesh] |  | 1,222,940 | 3:27:45 | 4/25/2026 |
| 4 | "Adolescent"[Mesh] |  | 2,399,857 | 3:27:36 | 4/25/2026 |
| 3 | #1 OR #2 |  | 33,321 | 3:26:29 | 4/25/2026 |
| 2 | gestational diabetes[tiab] OR GDM[tiab] OR diabetes mellitus, gestational[tiab] OR carbohydrate intolerance of pregnancy[tiab] | | 29,020 | 3:26:19 | 4/25/2026 |
| 1 | "Diabetes, Gestational"[Mesh] |  | 21,414 | 3:25:04 | 4/25/2026 |

**Table S2: Cochrane Library and Embase search query.**

| Search Name: | GDM |  |
| --- | --- | --- |
| Date Run: | 25/04/2026 15:09:05 |  |
| ID | Search | Hits |
| #1 | MeSH descriptor: [Diabetes, Gestational] explode all trees | 1836 |
| #2 | (gestational NEXT diabetes OR GDM OR "pregnancy induced diabetes" OR "pregnancy-induced diabetes" OR "maternal hyperglycemia"):ti,ab,kw | 4393 |
| #3 | #1 OR #2 | 4593 |
| #4 | (offspring OR children OR child OR infant OR adolescent OR young adult):ti,ab,kw | 427162 |
| #5 | MeSH descriptor: [Obesity] explode all trees | 23307 |
| #6 | MeSH descriptor: [Diabetes Mellitus, Type 2] explode all trees | 27733 |
| #7 | MeSH descriptor: [Insulin Resistance] explode all trees | 9990 |
| #8 | MeSH descriptor: [Metabolic Syndrome] explode all trees | 3782 |
| #9 | MeSH descriptor: [Hypertension] explode all trees | 26226 |
| #10 | MeSH descriptor: [Dyslipidemias] explode all trees | 9766 |
| #11 | (obesity OR overweight OR adiposity OR "body mass index" OR BMI OR "type 2 diabetes" OR T2DM OR hypertension OR "blood pressure" OR dyslipidemia OR "lipid disorder" OR "metabolic syndrome"):ti,ab,kw | 311987 |
| #12 | #5 OR #6 OR #7 OR #8 OR #9 OR #10 OR #11 | 323450 |
| #13 | long-term OR follow-up OR "follow up" OR observational OR prospective OR retrospective OR cohort OR longitudinal | 754638 |
| #14 | #3 AND #4 AND #12 AND #13 with Cochrane Library publication date Between Jan 2000 and June 2026 | 459 |

**Table S3:** Outcome data extracted from the included studies.

| studlab | Design | Sample size (I/C) | Age | Maternal age | Treatment | Gestational weight gain | BMI | % body fat | waist circumference | fasting glucose | 2h OGTT | HbA1c | HOMA-IR fasting insulin | SBP | DBP | total cholesterol | LDL-C | HDL-C | triglycerides (mmol/L or mg/dL) |
| --- | --- | --- | --- | --- | --- | --- | --- | --- | --- | --- | --- | --- | --- | --- | --- | --- | --- | --- | --- |
| Sun et al. 2020 | Observational cohort | 3172 women | 20-40 |  | not specified | 0.90 (0.68-1.18) | 2.01 (149-2.71) | NR | NR | NR | NR | NR | NR | NR | NR | NR | NR | NR | NR |
| Kusinski et al., 2025 | RCT | 428 (214/211) | ≥ 18 | 33.03 (5.04) | diet/lifestyle | NR | NR | NR | NR | NR | NR | 36.55 (4.62) vs 36.99 (3.3); -2.36 (-4.46 to -0.26) | NR | 117.9 (13.22) vs 118.36 (13.18) ; -0.96 (-3.50 to 1.58) | 72.40 (10.50) vs 70.92 (10.00); -0.77 (-1.27 to 2.81) | NR | NR | NR | NR |
| Nyen et al. 2024 | RCT | 118 | 31.2 (3.6) |  | diet/lifestyle | NR | NR | NR | NR | 0.17 (-0.011-0.35) | 0.024 (-0.027-0.075) | NR | NR | NR | NR | NR | NR | NR | NR |
| Bayoumi et al. 2021 | Observational cohort | 1260/3783 |  | 31.59 (5.87) | GDM | NR | 278/1463; 1.11 (0.83-1.48) | NR | NR | NR | NR | 5.28 (0.43) vs 6.19 (1.15) | NR | NR | NR | NR | NR | NR | NR |
| Morisset et al. 2011 | Retrospective | 294 (55/239) | 29.3 (4.7) |  | GDM | 3.40 (042) vs 1.87 (0.16); 1.25 (1.10-1.42) | NR | NR | NR | NR | NR | NR | NR | NR | NR | NR | NR | NR | NR |
| Rice et al. 2018 | Prospective | 981 (56) | 7 (6-8) |  | not specified | NR | 18.1 (16.7-19.7) vs 17.2 (17.0-17.4) | NR | 60.8 (57.2-64.8) vs 57.8 (57.2-58.4) | NR | NR | NR | 1.4 (1.0-2.0) vs 1.1 (1.1-1.2) | 100 (95-105) vs 99 (98-100) | 60 (56-63) vs 60 (59-60) | NR | NR | 51.9 (46.4-57.5) vs 52.4 (51.5-53.4) | 70.7 (57.6-86.8) vs 63 (60.8-65.3) |
| Hawkins et al. 2014 | RCT | 68 (33/35) | 18-40 | > 29 years: 24.2% | diet/lifestyle | 17.73 (1.0) vs 17.87 (0.59) | NR | NR | NR | 4.8 (8.8) vs 0.2 (0.5) | NR | NR | NR | NR | NR | NR | NR | NR | NR |
| Lapertosa et al. 2020 | Retrospective | 1870 recruited | 30.7 (6.7) | 29.3 (5.8) | not specified | NR | NR | NR | NR | NR | NR | NR | NR | NR | NR | NR | NR | NR | NR |
| Hanem et al. 2019 | RCT | 257 pregnant women (71/70) | 29.5 (3.9) |  | Metformin | NR | 17·8 (17·0-18·5) vs 16·8 (16·2-17·3); 1.02 (0.07-1.97) | 21·6 (18·9 to 24·4) vs 18·6 (16·3 to 20·9); 3·04 (−0·6 to 6·7) | 60·2 (58·3-62·1) vs 57·9 (56·5-59·4) ; 2·27 (−0·13- 4·67) | 4·69 (4·57- 4·82) vs 4·71 (4·60-4·83); −0·02 (−0·18- 0·15) | NR | 5·06 (5·01-5·10) vs 5·05 (4·98-5·11); 0·01 (−0·07-0·08) | 0·74 (0·63 to 0·86) vs 0·70 (0·59 to 0·79); 0·05 (-0·10 to 0·21) | 104 (102 to 106) vs 103 (101 to 105); 0·95 (−1·87 to 3·76) | 65 (64 to 67) vs 65 (63 to 67); 0·36 (1·97 to 2·69) | 4·14 (3·98 to 4·29) vs 4·15 (4·00 to 4·30); −0·01 (−0·23 to 0·20) | NR | 1·59 (1·51 to 1·68) vs 1·59 (1·49 to 1·70); 0·00 (−0·13 to 0·14) | 0·63 (0·57 to 0·68) vs 0·60 (0·53 to 0·66); 0·03 (−0·05 to 0·11) |
| Vinter et al. 2014 | RCT | 360 obese women (150/154) | 29 (27-32) |  | diet/lifestyle | NR | NR | NR | NR | 145/150, 4.9 (0.4) vs 5.0 (0.5) | 141/142, 6.4 (1.2) vs 6.5 (1.2) | NR | 0.7 (1.3) vs 1.0 (1.3), p = 0.02 | 123 (9), 142 vs 123 (11), 148 | 83 (9), 142 vs 84 (8), 148 | 6.5 (1.1), 146 vs 6.3 (1.0), 150 | 3.9 (1.0), 146 vs 3.6 (0.9), 149 | 1.8 (0.4), 146 vs 1.8 (0.4), 149 | 2.2 (0.6), 146 vs 2.4 (0.9), 149 |
| Davis et al. 2009 | Retrospective | 136 GDM | ≥ 30 | 30.7 (5.7) | GDM | NR | NR | NR | NR | 5.4 (1.2) vs 5.6 (1.4) | NR | 7.7 (1.9) vs 7.5 (1.4) | NR | NR | NR | NR | NR | NR | NR |
| Basu et al., 2021 | RCT | 34 | 27 (5.3) |  | diet/lifestyle | NR | NR | NR | NR | NR | NR | 4.5 (0.8) vs 5.2 (0.7) | 6.6 (5.8) vs 7.0 (3.3) | 116 (16) vs 123 (11) | 73 (8) vs 78 (7) | 227 (43) vs 216 (28) | 129 (35) vs 117 (23) | 62 (15) vs 61 (13) | 5.5 (2.2) vs 9.5 (6.6) |
| Wang et al., 2017 | RCT | 300 singleton women (150/150) | 32.1 (4.57) |  | Exercise | 8.38 (3.65) | NR | NR | NR | NR | 6.57 (1.18) vs 7.03 (1.62) | NR | 3.39 (1.19) vs 3.50 (1.11) | NR | NR | 5.82 (1.09) vs 5.81 (1.11) | 2.94 (0.82) vs 2.93 (0.84) | 1.72 (0.32) vs 1.65 (0.30) | 3.39 (1.19) vs 3.50 (1.11) |
| LeBlanc et al. 2021 | RCT | 326 (89/79) | 31.3 (3.5) |  | GDM screening | NR | NR | NR | NR | 81.2 (5.1) vs 84.8 (8.8) | NR | NR | 38/25, 1.7 (0.9) vs 2.5 (2.0) | NR | NR | NR | NR | NR | 38/25, 116.9 (48.2) vs 144.5 (79.9) |
| Fraticelli et al. 2018 | RCT | 80 GDM women (20 per arm) | 18-45 years | 35.0 (4.4) | Myo-inositol + D-Chiro-inositol | NR | NR | NR | NR | NR | NR | NR | 20/20 2.13 (1.02) vs 2.24 (1.16) | NR | NR | NR | NR | NR | NR |
| Clausen et al. 2009 | Observational cohort | 1066 offsrpings of women with GDM | 21.6 (1.8) | 29.5 (5.4) | diet/lifestyle | 10.55 (5.5) vs 13.7 (4.6) | 167/126; 2.09 (1.25-3.50) | NR | NR | ≥ 5.6 mmol/liter: 69/167 (41) vs 13/128 (10) | NR | NR | NR | 62/167 (37) vs 31/128 (24) | 6/167 (4) vs 2/128 (2) | NR | NR | 62/167 (37) vs 17/128 (13) | 26/167 (16) vs 6/128 (5) |

NR: Not reported.
